# Different effects of a perioperative single dose of dexamethasone on wound healing in mice with or without sepsis

**DOI:** 10.3389/fsurg.2023.927168

**Published:** 2023-04-11

**Authors:** Yuanyang Chen, Xiaoshan Chen, Quanhong Zhou

**Affiliations:** ^1^ Department of Anesthesiology, Affiliated Shanghai Sixth People's Hospital, Shanghai Jiao Tong University, Shanghai, China; ^2^ Department of ICU, Affiliated Shanghai Sixth People's Hospital, Shanghai Jiao Tong University, Shanghai, China

**Keywords:** dexamethasone, dose–response curve, macrophage, sepsis, wound healing

## Abstract

**Introduction:**

Sepsis delays wound healing owing to uncontrolled inflammation. A single perioperative dose of dexamethasone is widely used because of its anti-inflammatory effects. However, the effects of dexamethasone on wound healing in sepsis remain unclear.

**Methods:**

We discuss the methods to obtain dose curves and explore the safe dosage range for wound healing in mice with or without sepsis. A saline or LPS intraperitoneal injection was applied to C57BL/6 mice. After 24 hours, the mice received a saline or DEX intraperitoneal injection and full-thickness, dorsal wounding operation. Wound healing was observed by image record, immunofluorescence and histological staining. Inflammatory cytokines and M1/M2 macrophages in wounds were determined by ELISA and immunofluorescence, respectively.

**Results:**

Dose-response curves reflected the safe dosage range of DEX in mice with or without sepsis, from 0.121 to 2.03 mg/kg and from 0 to 0.633 mg/kg, respectively. we found that a single dose of dexamethasone (1 mg/kg, i.p.) promoted wound healing in septic mice, but delayed wound healing in normal mice. In normal mice, dexamethasone delays inflammation, resulting in an insufficient number of macrophages during the healing process. In septic mice, dexamethasone alleviated excessive inflammation and maintained the balance of M1/M2 macrophages in the early and late healing process.

**Discussion:**

In summary, the safe dosage range of dexamethasone in septic mice is wider than that in normal mice. A single dose of dexamethasone (1 mg/kg) increased wound healing in septic mice, but delayed it in normal mice. Our findings provide helpful suggestions for the rational use of dexamethasone.

## 1. Introduction

Wound healing is a well-orchestrated process involving many tightly controlled factors that work in concert (1). The healing process consists of four distinct yet overlapping stages: haemostasis, inflammation, proliferation, and remodelling. Many risk factors hamper the tight control of this process, including steroids, systemic inflammation status, and comorbidities.

Sepsis is characterised by a severe systemic inflammation due to an imbalance in the body’s response to infection (2, 3). Intra-abdominal infection has the highest mortality rate (30.7%) (4), and surgery is the most viable therapeutic measure to control such infections (5). However, sepsis has been proven in basic research and clinical trials to delay wound healing (6, 7), and patients with sepsis may suffer from impaired healing of the surgical incision.

Dexamethasone (DEX) has potent anti-inflammatory and immunosuppressive effects. The clinical outcomes of corticosteroid treatment in patients with sepsis or septic shock are associated with dosage. While low-dose dexamethasone has been reported to reduce mortality risk in patients with sepsis, high-dose dexamethasone may result in more harm than benefits (8). However, few studies have compared the effects of dexamethasone on wound healing in septic and non-septic conditions.

A perioperative single dose of dexamethasone is widely used to prevent postoperative nausea and vomiting (PONV) (9). However, the routine perioperative use of dexamethasone is still controversial (10, 11). Dexamethasone did not increase the incidence of surgical-site infection within 30 days after nonurgent noncardiac surgery (12) when compared with placebo controls. However, it is uncertain whether dexamethasone affects wound healing in the general surgical population (13).

Therefore, we hypothesised that dexamethasone (1 mg/kg, i.p.) may have different effects on wound healing in septic mice than in normal mice. We also explored the possible mechanism of action of dexamethasone in wound healing in the context of sepsis. Of note, we depicted the differences between the two dose–response curves, which explored the safe dosage range of dexamethasone in mice with or without sepsis.

## 2. Materials and methods

### 2.1. Dose–response curves

A total of 260 mice were allocated to two groups and received an intraperitoneal injection of dexamethasone at doses ranging from 0 to 5 mg/kg (9–11 doses per experiment). For normal mice, the complete range of dexamethasone doses was 0, 0.125, 0.25, 0.375, 0.5, 1, 1.25, 2.5, and 5 mg/kg (six mice per dose). Dexamethasone was administered to septic mice at 0, 0.125, 0.25, 0.5, 1, 1.25, 1.6, 2, 2.5, 3, and 5 mg/kg (6–10 mice per dose).

### 2.2. LPS sepsis model and medication procedure

Male C57BL/6 mice (6–8 weeks) were housed 3–5 per cage and had free access to water and food throughout the experiment; the padding was replaced at least three times a week. The protocol complied with the NIH Guide for the Care and Use of Laboratory Animals (NIH Publications No. 8023, revised 1978) and was approved by the Animal Ethics Committee of the Sixth People’s Hospital Affiliated to Shanghai Jiao Tong University, and was reported in accordance with the ARRIVE guidelines.

**Figure 1:**
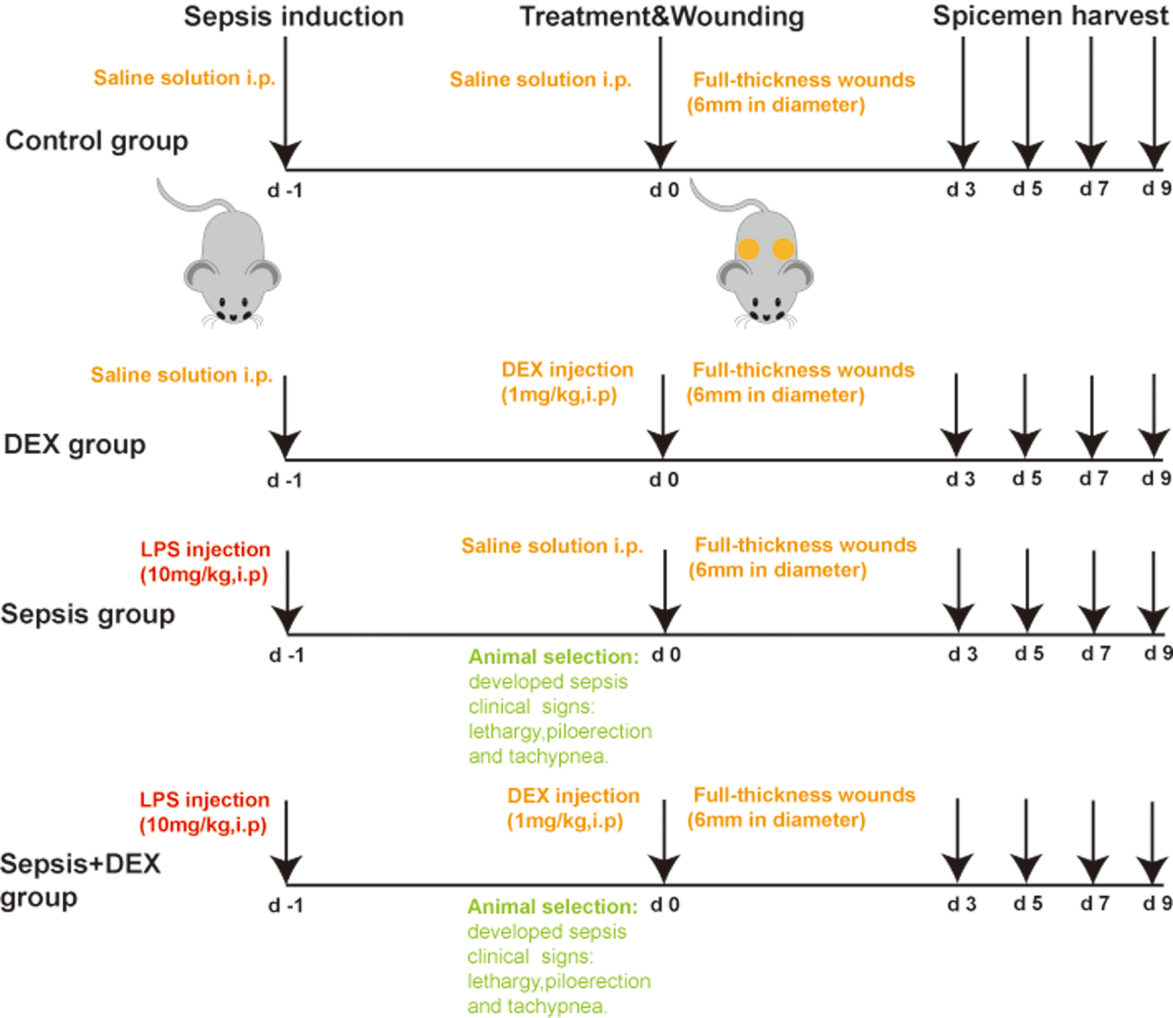
LPS sepsis model and medication procedure in the sepsis + DEX group. Medication procedure in 4 groups.

The mice were randomly separated into four experimental groups: control, DEX, sepsis, and sepsis + DEX group (Figure 1). The control and DEX groups received an intraperitoneal (i.p.) injection of saline solution, and the other groups received lipopolysaccharide (LPS) (O55:B5, Sigma Aldrich, St. Louis, MO, United States) injection (i.p.) at a dose of 10 mg/kg (14–17). After 24 h, mice with signs of lethargy, piloerection, and tachypnoea were diagnosed with sepsis (6). Dexamethasone (Sigma Aldrich, St. Louis, MO, United States), 1 mg/kg, was injected intraperitoneally into mice in the DEX group and sepsis + DEX group. The control and sepsis groups were injected with saline solution (i.p.).

### 2.3. Skin wound model and evaluation of wound closure

A mouse full-thickness wound model was generated and all the experiments were performed at 0, 3, 5, 7, and 9 days after the operation.

Before the experiment, the mice were anaesthetised by intraperitoneal administration of 3% pentobarbital sodium, and their back hair was removed. After that, their skin was disinfected with 75% alcohol. Two full-thickness wounds (6 mm diameter) were created on each side of the dorsal midline using a sterilised biopsy punch. The centres of the wounds were located 28 mm cranial from the beginning of the tail and 8 mm lateral to the spine.

Images of the wounds were captured using a digital camera. A ruler was used to standardise the measurements, and the results were quantified using ImageJ software (version 2.0.0, United States). The wound closure% was calculated using the equation (*A*_0 _− *A_n_*)/*A*_0_  ×  100, where *A*_0_ is the original wound area and *A_n_* is the wound area on days 3, 5, 7, or 9 post-surgery. At different time points, the mice were euthanised following the approved euthanasia protocol, and the original wound area and 2 mm-wide slices of skin around the wound surface were harvested and sectioned for further investigation.

### 2.4. Histological analysis

For morphometric analysis, the wound specimens were sectioned, stained with haematoxylin and eosin (H&E) according to the manufacturer’s protocol, and observed by light microscopy (Leica DM IL LED, Buffalo Grove, IL, United States). The thickness of granulation tissue was defined as the distance from the wounded dermal upper margin to the bottom of the area that is rich in cellular infiltration and revascularisation (18) (five measurement points per view). The thickness of the epidermis (imaged at ×200), granulation tissue (imaged at ×50), and the number of blood vessels (imaged at ×200) and follicles (imaged at ×50) were calculated in three randomly selected views per specimen using ImageJ software.

### 2.5. Immunofluorescence analysis

Immunofluorescence staining was performed on the tissue sections to assess vessel regeneration and granulation tissue with the expression of CD31 and α-smooth muscle actin, respectively. Cryosections of the tissue were fixed in paraformaldehyde (4%) for 15 min, washed with phosphate buffered saline (PBS) for 15 min, and blocked with 1% goat serum for 1 h before staining with primary antibodies overnight. The slices were incubated with the following primary antibodies: anti-mouse CD31 antibody (BD Pharmingen, clone 550274) 1:100 and anti-mouse α-smooth muscle actin (Cell Signalling Technology, clone 19245) 1:100.

To detect the effect of dexamethasone on macrophage polarisation in wounds, skin slices were processed by immunofluorescence staining with anti-mouse iNOS (Abcam, clone ab178945) 1:50 and anti-mouse Arg-1 (Santa Cruz Biotechnology, sc-271430) 1:500. After the primary antibodies were removed, Alexa Fluor 488- or Alexa Fluor 594-conjugated secondary antibodies (Cell Signalling Technology, MA, United States) 1:500 were used, followed by staining with DAPI (SouthernBiotech, Birmingham, United States).

### 2.6. Enzyme-linked immunosorbent assay

Wound samples collected on days 3, 5, 7, and 9 were frozen at −80°C. Frozen samples were carefully minced with sharp scissors and homogenised in cold PBS containing protease inhibitors (NCM Biotech, Suzhou, China) at a weight-to-volume ratio of 1 mg/mL. The samples were centrifuged at 2,500 rpm for 10 min at 4°C. The supernatant was analysed using a BCA Protein Assay Kit (Beyotime Biotechnology, Shanghai, China). Tissue levels of IL-6, TNF-α, and IL-10 were measured using enzyme-linked immunosorbent assay (ELISA) kits (Neobioscience Technology, Beijing, China), following the manufacturer’s instructions.

### 2.7. Statistics

Data are presented as the mean ± SEM. All statistical analyses were performed using Prism 9 (GraphPad, San Diego, CA, United States). For the comparison between normal and septic mice or between dexamethasone-treated normal mice and dexamethasone-treated septic mice, a two-way ANOVA followed by Tukey’s *post-hoc* test was used. For the safe dosage range of dexamethasone, the dose–response curve in normal mice was generated using a four-parameter nonlinear regression, and the curve in septic mice was generated using a bell-shaped nonlinear regression. Correlations of the dose–response curve in normal mice were assessed with the Pearson correlation coefficient. In all cases, statistical significance was set at *P* < 0.05.

## 3. Results

### 3.1. Dose curves of DEX for wound healing in mice with and without sepsis

It is challenging to obtain the dose–response curve of dexamethasone for wound healing. The dose variable is always quantitative, and the crux depends on the forms of the dependent variable. Table 1 shows a comparison of the response variables. In plan A, the dependent variable is the event incidence, whereas it is the relative wound closure% in plan B. Usually, the relationship between *x* and *y* in a dose–response curve is described by nonlinear regression models. Taking the four-parameter logistic regression model as an example [Equation (1)]

(1) $$y=l+\frac{u-l}{\left[1+exp[b(log(x)-log(e))]\right]}$$

where *y* is the response variable and *x* is the dose variable. If *y* decreases as the dose increases, *l* is the lower limit of the outcome variable at an infinitely large dose and *u* represents the upper limit of the outcome variable when the dose is zero. Parameter *e* indicates the dose inducing a response halfway between *u* and *l* parameters (19). In general (e.g., the dependent variable is cell viability), the value of *u* does not exceed 100% (the formula for cell viability determines the range of this value). Similarly, *l* was not less than 0% (20, 21). In plan A (Table 1), *u* and *l* satisfy the conditions in the model (*u *≤* *100% and *l *≥* *0%). There is only one new parameter to be defined: a certain value of the delayed-healing threshold. In our study, the average wound closure% of the control group was taken as the threshold value, which was used to normalise the data in experimental groups in previous studies (19, 20). However, *u* and *l* in plan B (the dependent variable was normalised by the control group) failed to meet the application conditions. Thus, Plan A (Table 1) was a better choice.

**Table 1:** Different doses of dexamethasone in normal mice (wound closure% on day 9).

	0 mg/kg	0.125 mg/kg	0.25 mg/kg	0.375 mg/kg	0.5 mg/kg	1 mg/kg	1.25 mg/kg	2.5 mg/kg	5 mg/kg
Wound closure% on day 9^a^	94.8 ± 4.73	96.9 ± 2.28	93.3 ± 1.39	92.5 ± 3.31	93.6 ± 2.16	85.9 ± 6.60	87.8 ± 3.17	87.4 ± 1.45	83.4 ± 5.06
Delayed-healing incidence	0/6	0/6	0/6	2/6	3/6	4/6	4/6	6/6	6/6

^a^ All values represent mean ± SD, *n* = 6.

**Figure 2:**
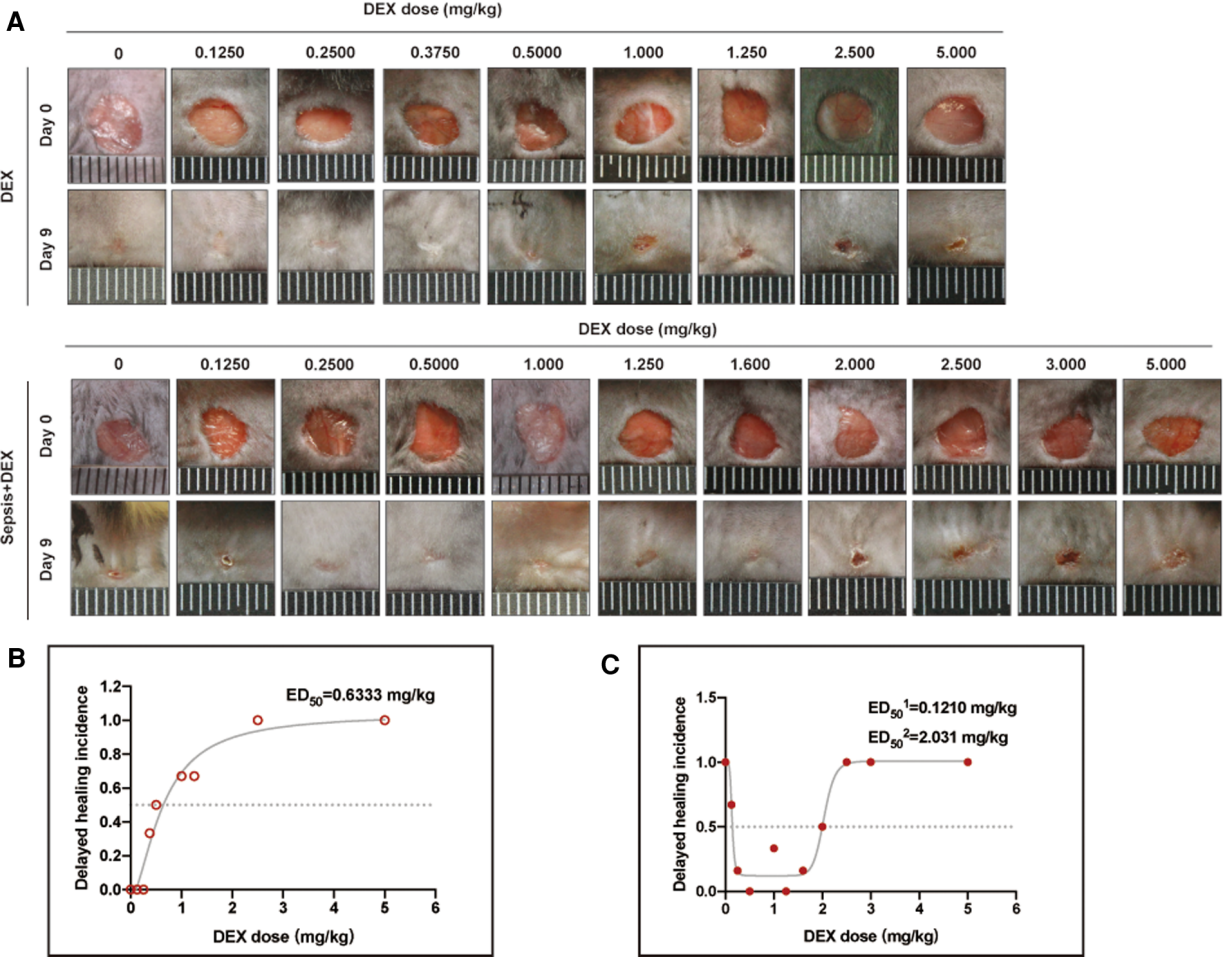
Dose curves of dexamethasone for wound healing in mice with and without sepsis. (**A**) Appearance of wounds on days 0 and 9 for DEX and sepsis + DEX groups. (**B**) Dose curve of dexamethasone for wound healing in normal mice. *R*^2^ is 0.959. (**C**) Dose curve of dexamethasone for wound healing in septic mice. *R*^2^ is 0.958. In (**B,C**), the non-healing wound was defined as that wound closure rate on day 9 was less than 90.1%. Parameter values are presented in Tables 2, 3.

To determine the association between dosing and the different effects of dexamethasone mentioned above, we performed a medium-safe dose experiment in mice with and without sepsis (Figure 2A). Taking the average wound closure% (94.8% ± 4.73%) of the control group on day 9 as the standard (when the wound closure% was less than this value, it was determined as delayed healing), we obtained the dose curves (Figures 2B,C). The dexamethasone dose at which delayed healing of 50% of the mice occurred was calculated as 0.633 mg/kg in dexamethasone-treated normal mice, while for dexamethasone-treated septic mice it ranged from 0.121 to 2.03 mg/kg (Figures 2B,C, Tables 2, 3). The safe dosage range for wound healing in mice without sepsis was from 0 to 0.633 mg/kg (Figure 2B), and that in mice with sepsis was from 0.121 to 2.03 mg/kg (Figure 2C). There was a positive correlation between the dose of dexamethasone and the incidence of delayed wound healing (*R*^2^ = 0.665, *P* = 0.007) in wounds without sepsis.

**Table 2:** Different doses of dexamethasone in septic mice (wound closure on day 9).

	0 mg/kg	0.125 mg/kg	0.25 mg/kg	0.5 mg/kg	1.0 mg/kg	1.25 mg/kg	1.6 mg/kg	2 mg/kg	2.5 mg/kg	3 mg/kg	5 mg/kg
Wound closure% on day 9^a^	85.5 ± 1.13	87.8 ± 3.50	93.6 ± 0.0447	97.2 ± 1.56	95.5 ± 5.19	93.7 ± 1.12	92.0 ± 1.37	90.1 ± 3.96	87.9 ± 1.88	86.4 ± 2.73	83.5 ± 7.25
Delayed-healing incidence	6/6	4/6	1/6	0/6	2/6	0/6	1/6	3/6	6/6	6/6	6/6

^a^ All values represent mean ± SD, *n* = 6.

**Table 3:** The data of wound closure%

Wound closure%	Control group	DEX group	Sepsis group	Sepsis + DEX
Day 3	62.4 ± 6.98	49.8 ± 5.68	31.9 ± 5.67	34.7 ± 7.86
Day 5	63.2 ± 5.48	58.4 ± 8.91	48.4 ± 3.56	33.5 ± 9.39
Day 7	80.0 ± 4.05	64.3 ± 6.53	78.2 ± 2.94	83.5 ± 3.59
Day 9	94.8 ± 4.38	85.9 ± 6.02	85.5 ± 1.06	95.5 ± 4.10

^a^ All values represent mean ± SEM, *n* = 6.

### 3.2. Intraperitoneal injection of dexamethasone (1 mg/kg) promoted wound healing in septic mice but delayed it in normal mice

**Figure 3:**
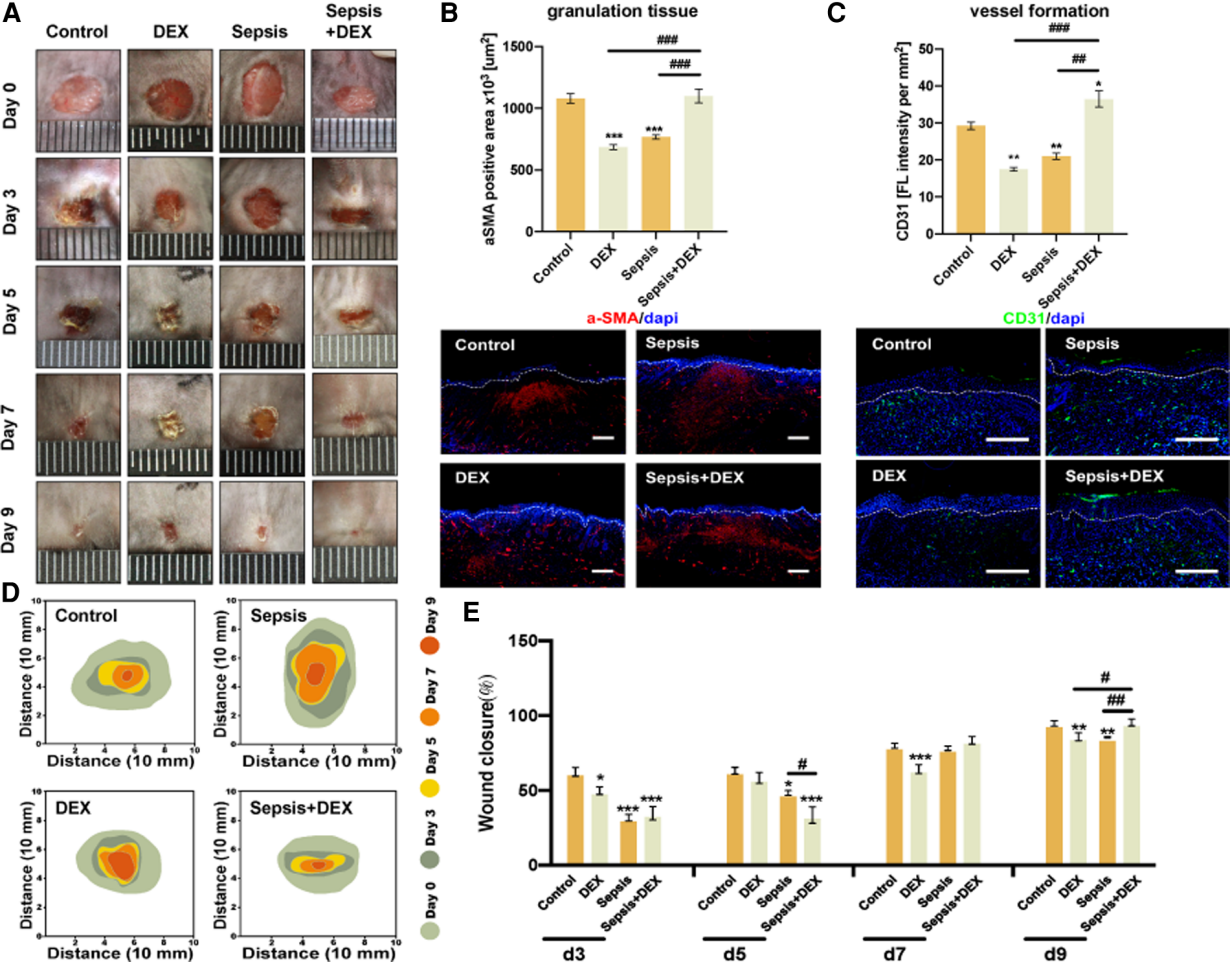
Intraperitoneal injection of dexamethasone (1 mg/kg) promoted wound healing in septic mice but delayed that in normal mice. Mice received intraperitoneal LPS (10 mg/kg) injection (sepsis and sepsis + DEX groups) or saline solution (control and DEX groups). After 24 h, dexamethasone was injected (1 mg/kg, i.p.) (DEX and sepsis + DEX groups) or normal saline (control and sepsis groups). Then, all groups had the wounding operation. Observation period included days 0, 3, 5, 7, and 9 after wounding. (**A**) Appearance of wounds on days 0, 3, 5, 7, and 9 for control, sepsis, DEX, and sepsis + DEX groups. (**B**) Granulation on day 9 after wounding. αSMA-IF (scale bar 500 μm), granulation tissue = αSMA-positive area (mean ± SEM, *n* = 6 per group). (**C**) Vessel formation on the 9th day after wounding. CD31-IF (scale bar 500 μm), angiogenesis = CD31 distribution/wound area (mean ± SEM, *n* = 3 per group). (**D**) Schematic diagram of wound area during 9 days for control, sepsis, DEX, and sepsis + DEX groups. Visualised by Adobe Illustrator. (**E**) Wound closure for each group. Quantitative data of image records (mean ± SEM, *n* = 6 per group). Wound closure rate was measured by the equation: (*A*_0 _−_ _*A_n_*)/*A*_0_  ×  100, where *A*_0_ is the wound area on day 0, and *A_n_* is the wound area of days 3, 5, 7, or 9 post-wounding. *P < 0.05, **P < 0.01, ***P < 0.001, vs. control group; #*P* < 0.05, ##*P* < 0.01, ###*P* < 0.001, vs. other groups. IF, immunofluorescence; αSMA, α-smooth muscle actin.

To explore whether a single dose of dexamethasone (1 mg/kg, i.p.) has different effects on wound healing in normal and septic mice, we compared wound states among four groups of mice (six mice per group): control (intraperitoneal injection of saline solution to C57BL/6 mice), DEX (DEX, i.p.), sepsis (LPS, i.p.), and sepsis + DEX (septic mice with DEX, i.p.) groups. As shown in Figure 3, dexamethasone delayed wound healing from day 3 after wounding in normal mice but accelerated the healing process in septic mice from day 7 (Figure 3E), which was visualised by image records and a schematic diagram (Figures 3A,D). By immunofluorescence staining, we observed less granulation tissue (revealed by αSMA-positive areas) and blood vessels (indicated by CD31 distribution) in the DEX group on day 9 after wounding than in the control group (Figures 3B,C). In contrast, more granulation tissue and blood vessels were seen in the sepsis + DEX group than in the sepsis group.

### 3.3. Intraperitoneal injection of dexamethasone (1 mg/kg) improved tissue regeneration in septic mice

**Figure 4:**
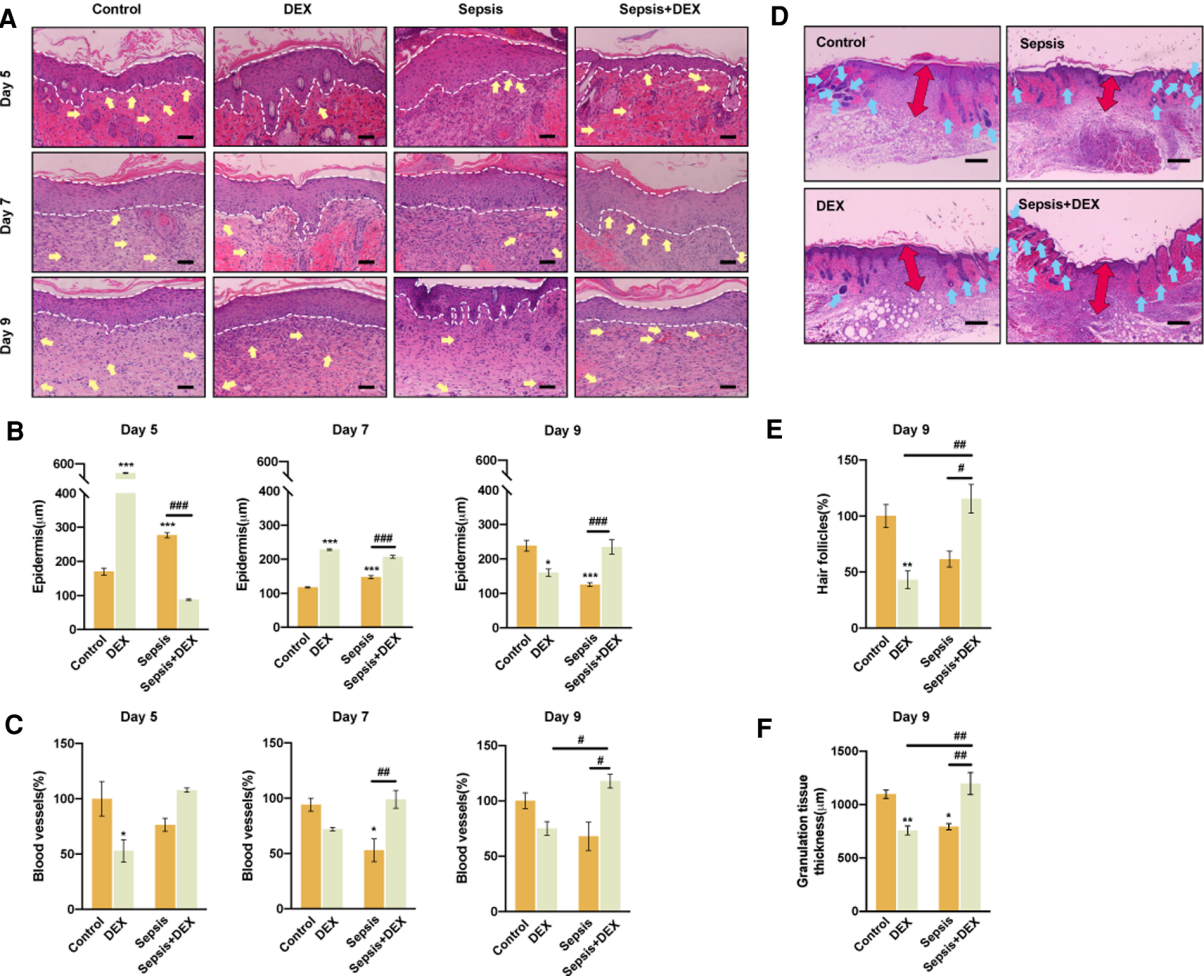
Intraperitoneal injection of dexamethasone (1 mg/kg) improved tissue regeneration in septic mice. (**A**) Wound H&E staining for control, sepsis, DEX, and sepsis + DEX on the 5^th^, 7^th^, and 9^th^ day (blood vessels: yellow arrows, hair follicles: blue arrows, boundary of epithelium: white lines). Scale bar: 100 µm. (**B**) Epidermis thickness assessments for different groups on the 5^th^, 7^th^, and 9^th^ day. (**C**) Blood vessels regeneration on the 5^th^, 7^th^, and 9^th^ day. Quantified data were presented by relative number percentage. Control group on 5^th^ day was set as 100%. (**D**) Granulation tissue (red arrows) thickness and amount of hair follicles (blue arrows) for different groups on the 9^th^ day. Scale bar: 500 µm. (**E**) Hair follicles for different groups on day 9 post-wounding. Data were shown in relative number percentage. Control group was set as 100%. (**F**) Quantitative data of granulation tissue thickness. Data are shown as mean ± SEM, *n *= 3.*P < 0.05, **P < 0.01, ***P < 0.001, vs. control group; #*P* < 0.05, ##*P* < 0.01, ###*P* < 0.001, vs. other groups.

Next, we assessed the quality of the regenerated tissue in the wounds by histological analysis (Figure 4). Mice in the DEX group expressed thinner epidermis on day 9 than those in the control group (Figures 4A,B). However, wounds in the sepsis + DEX group had thicker epidermis than those in the sepsis group, which indicated that dexamethasone improved epidermal regeneration in septic mice.

Soon after the injury, the fibrin clot is replaced by blood vessel-rich granulation tissue. In general, new vessel formation is essential for tissue repair by delivering nutrients and oxygen to injured tissue and removing waste products and carbon dioxide (22). Thus, blood vessel and granulation tissue conditions are closely related to the healing situation and are often used as evaluation parameters (23, 24). During the entire observation period, dexamethasone promoted angiogenesis and granulation tissue formation in wounds of the sepsis + DEX group, exhibiting a larger number of blood vessels and thicker granulation tissue than those in the sepsis group. However, dexamethasone suppressed vessel regeneration in the DEX group (Figures 4A,C).

The hair follicle is another regeneration parameter (as it is rich in several kinds of skin stem cells, which improves wound healing in re-epithelialisation and angiogenesis) (25–28), which was significantly increased by dexamethasone in the sepsis + DEX group compared with that in the DEX group on day 9 (Figure 4E). These results demonstrate that dexamethasone improved wound healing only in septic mice, but not in normal mice, in terms of enhancing angiogenesis and the formation of granulation and follicles.

### 3.4. Intraperitoneal injection of dexamethasone impaired the initiation of inflammation in wounds of normal mice but not in septic mice

Accumulating evidence shows that the effect of dexamethasone on wound healing is accompanied by changes in inflammatory cytokines (29–31). To reveal the association between the inflammatory response and the different effects of dexamethasone (1 mg/kg, i.p.) on wound healing in septic and normal mice, we assessed the expression of pro-inflammatory cytokines (IL-6 and TNF-α) and anti-inflammatory cytokine (IL-10) in the wound healing process (3–9 days).

**Figure 5:**
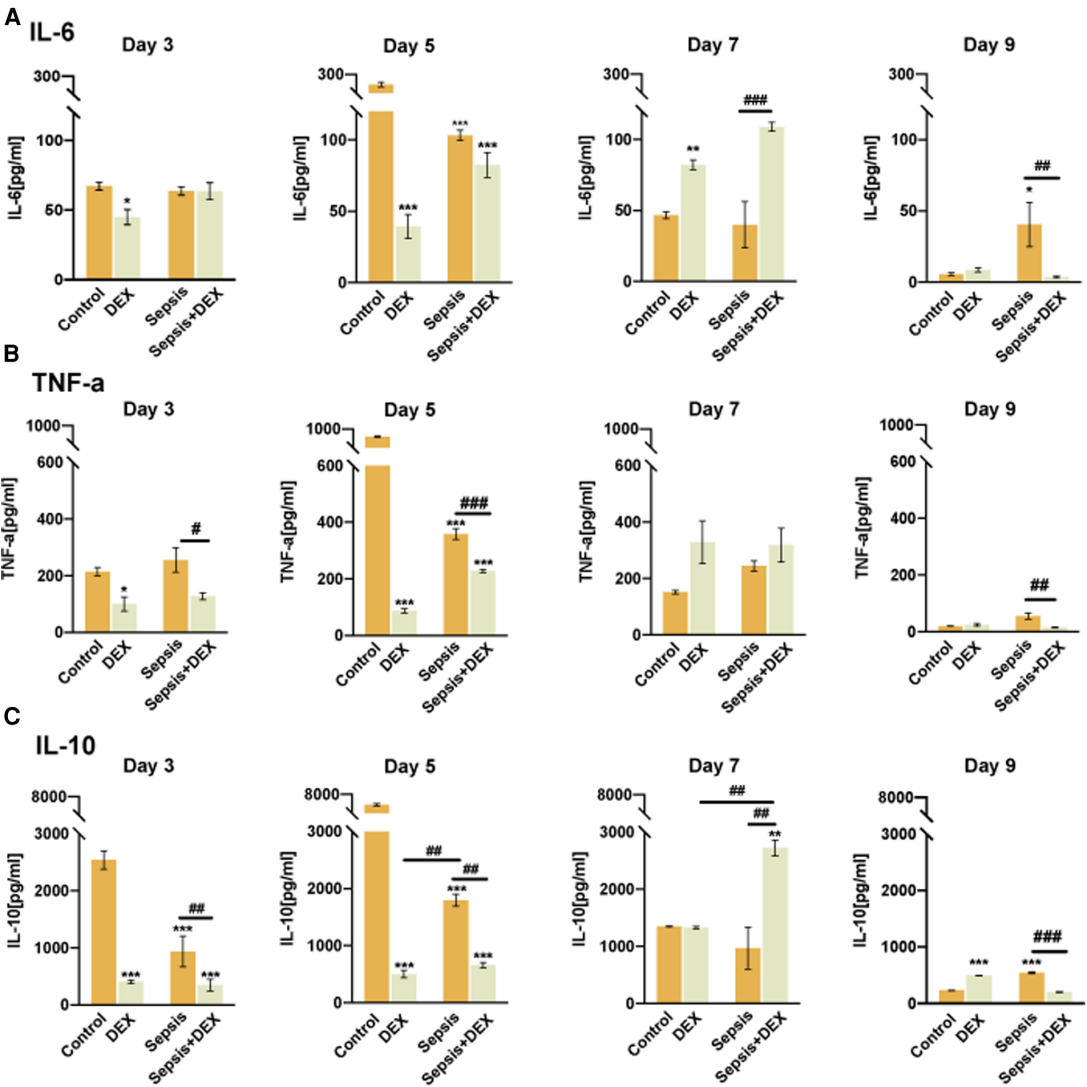
Intraperitoneal injection of dexamethasone impaired the initiation of inflammation in wounds of normal mice but not in septic mice. ELISA analysis of IL-6, TNF-α, and IL-10 in wounds. (**A**) The concentration of IL-6 in skin defects among control, DEX, sepsis, and sepsis + DEX groups on the 3^rd^, 5^th^, 7^th^, and 9^th^ days after wounds creation. (**B**) TNF-α expression in tissue among the four groups on the 3^rd^, 5^th^, 7^th^, and 9^th^ day after wounding. (**C**) IL-10 expression of wounds among different groups on the 3^rd^, 5^th^, 7^th^, and 9^th^ day after the operation. All values represent mean ± SEM, *n* = 3.*P < 0.05, **P < 0.01, ***P < 0.001, vs. control group; #*P* < 0.05, ##*P* < 0.01, ###*P* < 0.001, vs. other groups. TNF-α, tumour necrosis factor-α; ELISA, enzyme-linked immunosorbent assay.

As shown in the ELISA analysis (Figure 5), early in wound healing (days 3 and 5), dexamethasone decreased the expression of inflammatory cytokines in the DEX group (compared to the control group) and the sepsis + DEX group (compared to the sepsis group). Later in the healing process, the DEX group showed persistent inflammation with a higher level of IL-6 on day 7 than that in the control group. In addition, more TNF-α was secreted in the DEX group on days 7 and 9, although there were no statistically significant differences between the DEX and control groups. These results were different in the sepsis + DEX group. Dexamethasone suppressed the secretion of pro-inflammatory cytokines (IL-6 and TNF-α) during the early stages of wound healing. A higher level of IL-6 on day 7 and a lower level of inflammation (both IL-6 and TNF-α) on day 9 were observed in the Sepsis + DEX group than in the sepsis group.

Of note, IL-10 showed a trend of changes, which were lower on days 3 and 5 in the DEX-treated groups than in the saline groups, and then reached a peak on day 7 but declined again on day 9.

### 3.5. The initiation of inflammation and subsequent macrophage polarisation towards *M*2 relied on adequate *M*1 macrophages in early wound healing

**Figure 6:**
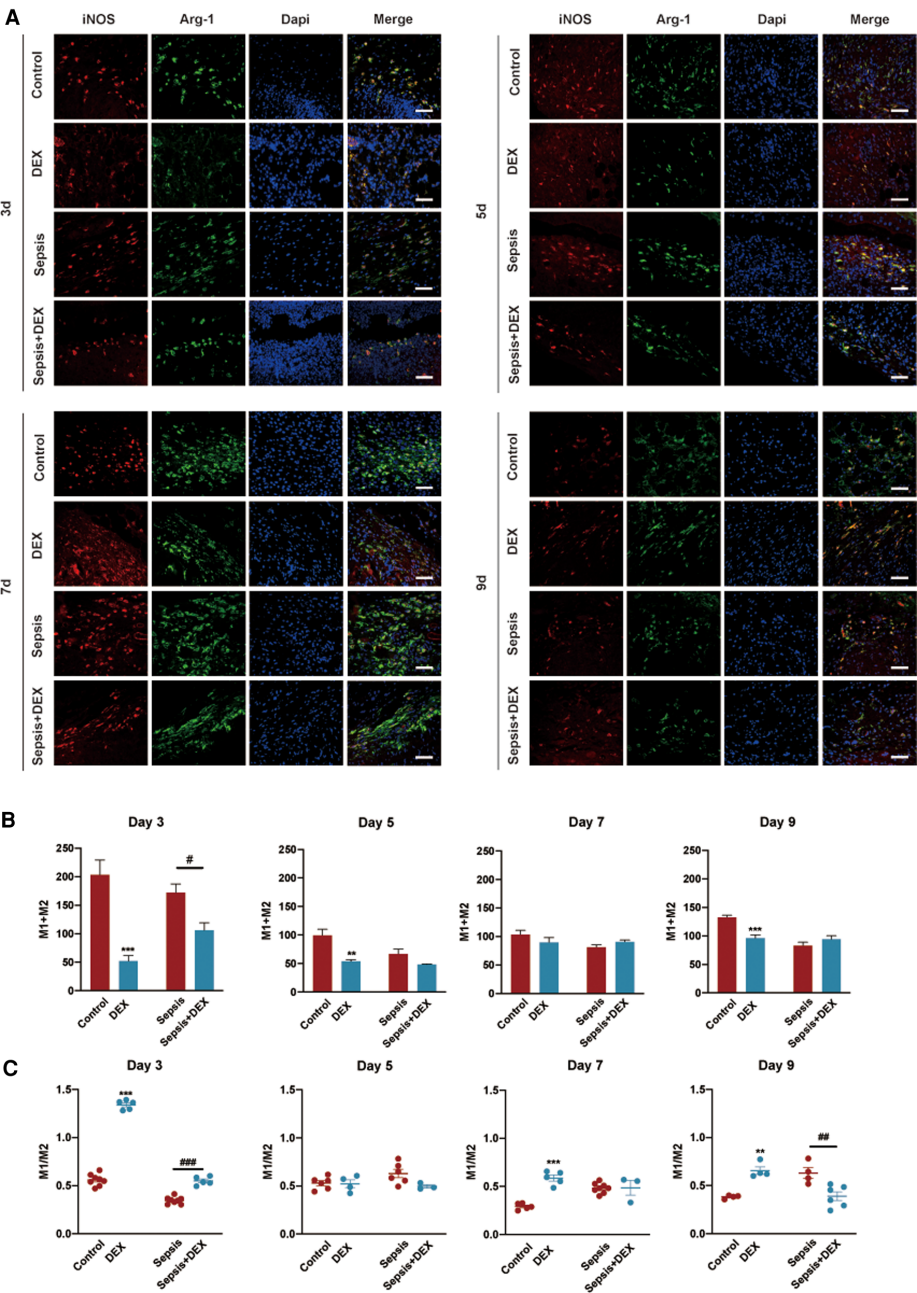
The initiation of inflammation and subsequent macrophage polarisation towards *M*2 relied on adequate *M*1 macrophages in early wound healing. Immunofluorescence staining analysis of *M*1 and *M*2 macrophages in wounds in various groups on the 3^rd^, 5^th^, 7^th^, and 9^th^ day. (**A**) Representative histological slices of wounds stained by immunofluorescence: red (iNOS, *M*1 macrophages marker), green (Arg-1, *M*2 macrophages marker), and blue (DAPI). Scale bar: 100 µm. (**B**) Number of *M*1 and *M*2 macrophages. (**C**) *M*1/*M*2 ratio. (**B,C**) are quantised data of (**A**). All values represent mean ± SEM, *n* > 3.*P < 0.05, **P < 0.01, ***P < 0.001, vs. control group; #*P* < 0.05, ##*P* < 0.01, ###*P* < 0.001 vs. sepsis group. iNOS, inducible nitric oxide synthase; Arg-1, arginase-1.

Macrophages play a prominent role in tissue repair by balancing the pro- and anti-inflammatory responses. Early in wound repair, *M*1 macrophages participate in pro-inflammatory activities and have traditionally been marked with iNOS. *M*2 macrophages, which promote inflammation resolution and tissue repair during the later stages of wound healing, are marked with Arg-1. To study how dexamethasone affects inflammation progression, we checked the number of macrophages (*M*1 + *M*2) and the *M*1/*M*2 ratio in wound sites using immunofluorescence staining (Figure 6).

**Figure 7:**
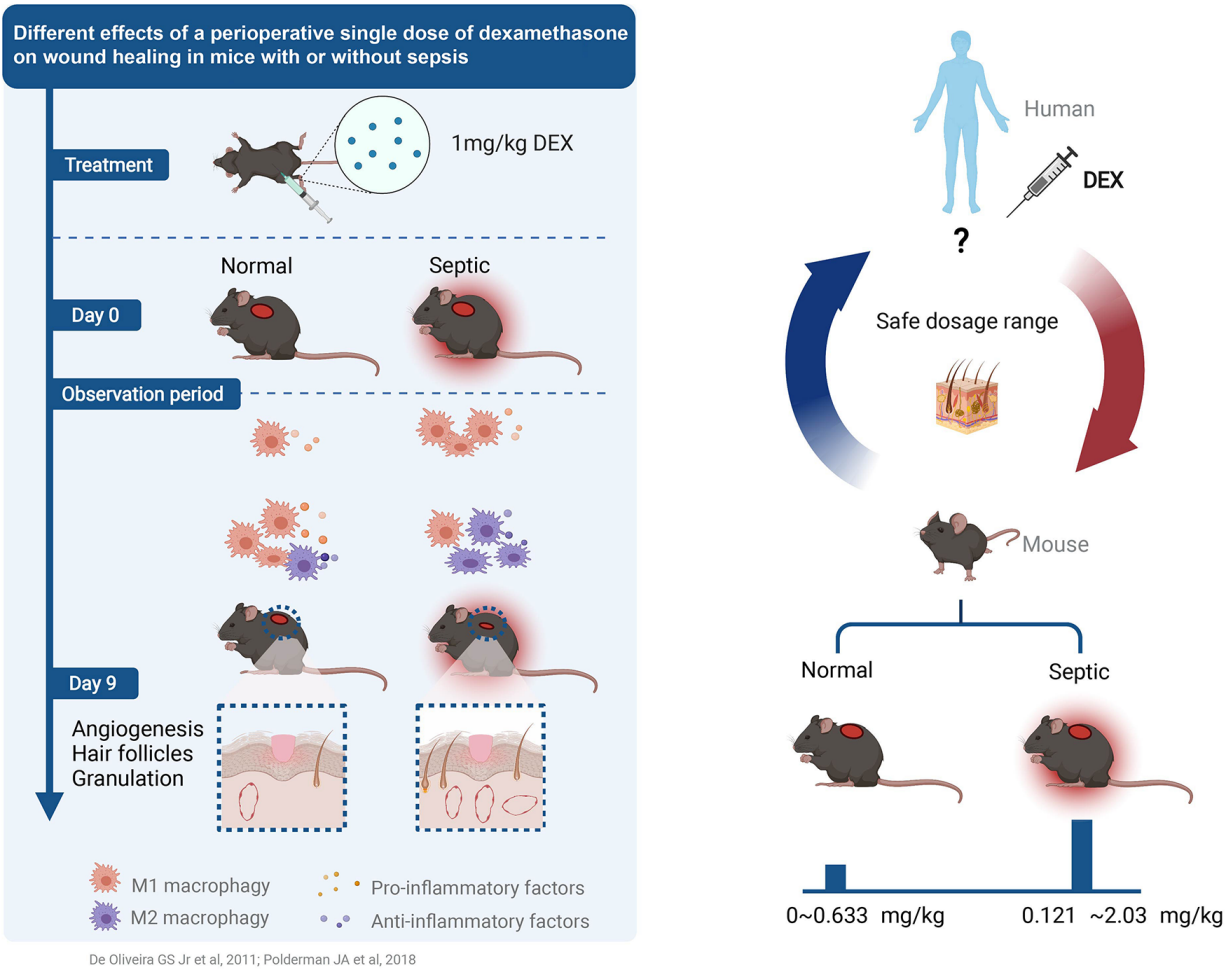
Different effects of a perioperative single dose of dexamethasone on wound healing in mice with or without sepsis (50, 51).

Throughout the wound healing process, mice in the DEX group had fewer macrophages (*M*1 + *M*2) than those in the control group. On day 3, more macrophages were observed in the sepsis group, but fewer were observed in the sepsis + DEX group. There were no significant differences in the number of *M*1 + *M*2 macrophages between the sepsis and Sepsis + DEX groups on days 5, 7, and 9.

The wounds in the DEX group displayed a higher *M*1/*M*2 ratio than those in the control group on day 3. During later tissue repair, a higher *M*1/*M*2 ratio was observed in the DEX group than in the control group.

On the other hand, there was a higher *M*1/*M*2 ratio in wounds of the sepsis + DEX group than in the sepsis group on day 3. Wounds in the sepsis + DEX group had a lower *M*1/*M*2 ratio on day 9 than those in the sepsis group.

These results indicate that dexamethasone decreased *M*1 macrophages in early wound healing, which may result in the phenotypic switch of macrophages from *M*1 to *M*2 being delayed or failing to occur.

## 4. Discussion

Dexamethasone, an approved corticosteroid medication, is widely used for prophylaxis and treatment of PONV. However, the safe dosage of dexamethasone for wound healing in the perioperative period, particularly in patients with sepsis, remains unknown.

Herein, we found that the differences between normal and septic mice were dose-related and described them in the dose–response curves. By comparing the incidence of delayed healing, we demonstrated that the therapeutic window of dexamethasone in septic mice was broader. Identification of the relationship between inflammatory response and wound healing may provide a plausible explanation for dose-related discrepancies.

Significantly, these curves may be beneficial for a rational plan of dexamethasone use. The differences between them highlight some important considerations regarding perioperative dexamethasone administration, namely, dosing and disease of the body.

By observing the dose–response curves, we found that intraperitoneal injection of dexamethasone (1 mg/kg) played a positive role in wound repair in septic mice but impaired it in normal mice. Previous studies have focused on either the association of wound healing and sepsis (6) or wound healing and dexamethasone (32); however, few investigations have focused on the impact of single-dose dexamethasone on wound repair under septic status. This is, so far as we know, the first study to explore the single dose of dexamethasone (1 mg/kg, i.p.) on wound healing in mice with and without sepsis and found the different effects of dexamethasone. Durmus et al. (32) identified that a single dose of dexamethasone at 1 mg/kg may have negative effects on wound healing in a prospective, randomised, experimental rat model. Our experiments confirmed these findings, and we found that a low dose of dexamethasone may benefit wound healing in septic mice.

Here, we monitored the progression of wound healing in two aspects: wound performance and the quality of tissue regeneration, and evaluated them using diverse parameters: wound closure, epidermal proliferation, angiogenesis, and the formation of granulation tissue and follicles. Moreover, we compared the results with those of their septic counterparts and found different effects of dexamethasone, which were visualised by multiple experimental methods: image recording, H&E staining, and immunofluorescence staining.

The dose–response curve in normal mice was generated using a four-parameter nonlinear regression; Carvalho et al. (33) and Ginosar et al. (34) used this model to describe the relationship between dose and response in dose–response curves, and correlations were assessed with the use of linear regression in their studies. For wounds without sepsis, there was a positive correlation between the dose of dexamethasone and the incidence of delayed wound healing in our study. The curve in septic mice was generated using a bell-shaped nonlinear regression; Owen et al. (35) and Zhu et al. (36) used this model to describe the relationship between dose and response in dose–response curves. They performed statistical analyses of the dose–response curves, However, the forms of the response in our study are different from theirs, and the bell-shaped model is the sum of two dose–response curves; the relationship between the dose of dexamethasone and the incidence of delayed wound healing is not suitable to describe with simple positive/negative correlation. If we perform statistical analyses of the dose–response curves, the other form of the response (relative wound closure rate) will be chosen, which may produce more problems that we discuss in Table 4.

**Table 4:** Selection of the dependent variable in dose–response curves.

	The dependent variable is the event incidence (A)	The dependent variable is relative wound closure rate (B)	The better one
Supporting evidence^a^	Yes	No	A
Data	Transformed	Transformed	—
Curve-fitting model	Yes	Yes	—
Number of new parameters	1	2	A
The literature support of new parameter(s)	Yes	No	A

^a^ Carvalho et al. (33) and Ginosar et al. (34) have defined the event incidence as the dependent variable in the dose–response curve.

The process of wound healing involves a programmed local inflammatory reaction that requires precise coordination and is dictated mainly by macrophages in response to tissue damage. Early in wound healing, the initial inflammatory response is characterised by macrophage tissue destruction, production of inflammatory cytokines, and clearance of pathogens and debris. This is followed by the resolution of inflammation and initiation of tissue repair. The timing of both initiation and resolution of inflammation is essential for restoring tissue integrity after injury. Macrophages, which initiate and resolve inflammation, play an indispensable role in wound healing and contribute to tissue regeneration. The transition from pro-inflammatory to reparative phenotypes is required for effective wound healing in an orderly manner (32, 37). However, in the pathological status, the phenotypic switch of macrophages can be perturbed, resulting in the insufficiency and imbalance of pro-inflammatory and anti-inflammatory macrophages, preventing wound repair (36, 38, 39).

Our study demonstrated that a single dose of dexamethasone reduced pro-inflammatory cytokine levels and the number of macrophages in the wounds of normal mice in the early healing stage. This failure to generate an initial inflammatory response negatively impacts the downstream orchestration of subsequent phases, resulting in ongoing inflammation instead of proceeding into the proliferative phase (39, 40).

Our data suggest that dexamethasone impairs the initiation of inflammation in normal mice by reducing the number of macrophages, which may result from the inhibition of macrophage polarisation towards the *M*1 phenotype.

Unlike in normal mice, dexamethasone decreased the inflammatory response in the early healing of septic mice, and in the later stage, the wounds expressed a lower level of inflammatory cytokines. During the wound healing process, the total number of *M*1 and *M*2 macrophages was equal in the sepsis and sepsis + DEX groups. However, we found that there were sufficient *M*1 macrophages in the early stages and *M*2 macrophages in later wound healing in dexamethasone-treated septic mice, exhibiting a higher *M*1/*M*2 ratio and a lower *M*1/*M*2 ratio, respectively. This ensures that macrophages function in both pro-inflammatory and reparative phenotypes (41–43). These data indicate that dexamethasone alleviated the degree of inflammation rather than impairing the initiation of inflammation in wounds of dexamethasone-treated septic mice in the early stages; thus, healing progressed with resolving inflammation.

During wound healing, the local inflammatory response to injury may be influenced by dysregulation of systemic inflammation. In the current study, we found that dexamethasone reduced excessive inflammation by reducing the number of macrophages but maintained the balance between *M*1 and *M*2 macrophages in wounds in the context of sepsis. This may be because of the following reasons. First, DEX may disturb macrophage polarisation in wounds. Indeed, using *in vitro* and *in vivo* models, prior studies have demonstrated that glucocorticoids have an inhibitory effect on the polarisation of macrophages towards a pro-inflammatory phenotype (44–46). Second, DEX may perturb the migration of macrophages from the circulation; however, this hypothesis contradicts the observations of Chatzopoulou et al. (47) and Xie et al. (44) that glucocorticoids have a limited effect on the migration of macrophages in the zebrafish tail amputation model.

In the peritonitis model, a systemic inflammatory response occurs with large numbers of *M*1 macrophages (44, 48), which may provide a pre-inflammatory environment. After wounding, large numbers of monocytes are recruited from circulation into the wound bed, and such monocytes may differentiate into pro-inflammatory macrophages in response to the pre-inflammatory environment. Dexamethasone treatment, however, reduced the expression of inflammatory cytokines and the polarisation of macrophages in early stages (29, 49) and had limited effects on specific macrophages (pre-inflammatory environment maker), which maintained the coordination of the initiation and resolution of inflammation in wound healing. However, the effect of dexamethasone may be conditional, and further investigation is warranted.

Our study offers insight into the rational use of dexamethasone. However, this study has some limitations that must be addressed. Since we know that the effects of DEX on wound healing are associated with macrophages, further studies are necessary to clarify the regulatory mechanism by which DEX affects phenotypic changes in macrophages during the wound healing process in sepsis. In addition, rodent models have limitations in the evaluation of the effects of drugs in clinical practice and do not completely reflect human disease; thus, more forms of disease models and injections should be investigated.

In conclusion, wound healing was promoted in septic mice but delayed in normal mice following a single-dose intraperitoneal injection of dexamethasone. Dexamethasone reduced inflammation without impairing the *M*1-induced initiation of inflammation and polarisation towards *M*2 in septic mice but impaired inflammation in normal mice. The two curves reflect the dose-related differences, which may provide considerable suggestions for the rational use of dexamethasone.

## Data Availability

The original contributions presented in the study are included in the article/Supplementary Material, further inquiries can be directed to the corresponding author/s.
